# The Influence of Foveal Lexical Processing Load on Parafoveal Preview and Saccadic Targeting During Chinese Reading

**DOI:** 10.1037/xhp0000644

**Published:** 2019-06

**Authors:** Manman Zhang, Simon P. Liversedge, Xuejun Bai, Guoli Yan, Chuanli Zang

**Affiliations:** 1 Academy of Psychology and Behavior, Tianjin Normal University; 2 School of Psychology, University of Central Lancashire; 3 Academy of Psychology and Behavior, Tianjin Normal University; 4 Academy of Psychology and Behavior, Tianjin Normal University, and School of Psychology, University of Central Lancashire

**Keywords:** Chinese reading, eye movements, foveal load, parafoveal preview, saccadic targeting

## Abstract

Whether increased foveal load causes a reduction of parafoveal processing remains equivocal. The present study examined foveal load effects on parafoveal processing in natural Chinese reading. Parafoveal preview of a single-character parafoveal target word was manipulated by using the boundary paradigm (Rayner, 1975; pseudocharacter or identity previews) under high foveal load (low-frequency pretarget word) compared with low foveal load (high-frequency pretarget word) conditions. Despite an effective manipulation of foveal processing load, we obtained no evidence of any modulatory influence on parafoveal processing in first-pass reading times. However, our results clearly showed that saccadic targeting, in relation to forward saccade length from the pretarget word and in relation to target word skipping, was influenced by foveal load and this influence occurred independent of parafoveal preview. Given the optimal experimental conditions, these results provide very strong evidence that preview benefit is not modulated by foveal lexical load during Chinese reading.

## Public Significance Statement

The findings of the present study show that foveal processing load, as manipulated through lexical frequency, has no modulatory influence on preview benefit for the subsequent word in a sentence during natural Chinese reading. Foveal processing difficulty does, however, influence saccades that determine where the eyes fixate next, and this influence occurs regardless of preview benefit for the following word. These results pertain directly to the Foveal Load Hypothesis that has received significant scrutiny in recent years. They also directly inform current understanding of the complexities of the relationship between the oculomotor control system that is responsible for positioning the point of fixation during reading and the complex cognitive processes that occur during written Chinese language comprehension.

Every time a reader moves their eyes, the visual information delivered to the brain derives from not only the fixated word in the fovea, but also from nonfixated words in the parafovea. A wealth of studies have demonstrated consistently that preview of parafoveal words facilitates processing of that text when it is fixated, reducing fixation time. This effect has been referred to as preview benefit (see Rayner, 1998, 2009, for reviews) and can be measured by using an eye contingent display change technique termed the boundary paradigm (Rayner, 1975). In the boundary paradigm, prior to direct fixation, a target word embedded in a sentence is replaced by some form of preview and an invisible boundary is positioned immediately prior to the word. When the reader makes a saccade that crosses the boundary, the preview is changed to the target word. The reader remains unaware of the change because it occurs during a saccade when clear visual input is unavailable. Using this approach, it is possible to manipulate the relationship between the preview and the target word. If the preview facilitates processing of the target (through reduced fixation durations on the target) then preview benefit is said to have occurred, demonstrating that readers were able to extract information from the parafovea reflecting the common characteristics that the preview and target share. Thus, the boundary paradigm allows researchers to explore quite directly the manner in which parafoveal text is processed prior to direct fixation.

An issue that remains equivocal to date concerns whether parafoveal processing is influenced by the difficulty of current foveal processing. The classic study conducted by Henderson and Ferreira (1990; see also Rayner, 1986) demonstrated that preview benefit was reduced under conditions of increased foveal processing difficulty. Specifically, Henderson and Ferreira manipulated a pretarget word such that foveal processing load was high or low via a lexical and a syntactic manipulation in two boundary paradigm experiments. Additionally, the parafoveal preview of the target word was manipulated such that it was identical, visually similar or dissimilar. Henderson and Ferreira found that preview benefit for the target word was reduced when the pretarget word was infrequent or caused syntactic processing difficulty, and they concluded that the extent of the perceptual span was reduced under conditions of increased foveal processing load. This conclusion was in line with that formed in an earlier study by Rayner (1986). In his experiment, Rayner used the moving window paradigm to measure the spatial extent of the perceptual span in the children of different grades, as well as adult skilled readers. He found that beginning readers (i.e., less skilled readers) had a reduced perceptual span relative to skilled readers, and importantly, in his fourth experiment in which he tested fourth grade readers, he showed that the size of perceptual span was reduced for text that was categorized as difficult relative to text that was categorized as easy. While Rayner’s results and conclusions seem clear, it is important to note that the boundary paradigm that Henderson and Ferreira used to explore parafoveal processing in relation to foveal load is ordinarily used to investigate the kind of visual and linguistic information that is extracted from upcoming parafoveal words, rather than the spatial extent of parafoveal processing. To this extent, it may be the case that Rayner’s study and findings led Henderson and Ferreira to form similar conclusions, even though, perhaps, the boundary paradigm they adopted more likely provided a measure of the degree to which the parafoveal word was effectively processed (i.e., the size of any preview benefit) rather than the spatial extent of such processing. However, recently Luke (2018) used the moving window paradigm to examine how foveal lexical properties (i.e., word frequency or word predictability) influence parafoveal word processing during paragraph reading. Two preview window conditions were created: the window was either restricted to the currently fixated word N (no preview condition), or extended to word N + 1 (preview condition). For both conditions, two words were always visible to the left of the currently fixated word N. Consistent with Henderson and Ferreira, Luke found foveal load interacted with preview. Specifically, the target word was fixated for less time when the pretarget word was more frequent than when it was less frequent, and such an effect occurred only when a preview of the target word was available. Regardless of the specific manipulations and conclusions formed on the basis of these studies, the important point is that Henderson and Ferreira, and Rayner raised the very interesting theoretical possibility that foveal processing load—that is, the difficulty of the fixated word—might have some kind of influence on the nature of parafoveal processing. The current study aimed at exploring this possibility in Chinese reading. Before providing more details about the present experiment, we will first consider some other relevant experimental studies.

White, Rayner, and Liversedge (2005a) conducted a study that was similar to that of Henderson and Ferreira (1990). They used the boundary paradigm to examine whether preview benefit for an upcoming target word was reduced when the pretarget word was low frequency. They used shorter target words than Henderson and Ferreira and investigated whether the reader’s awareness of eye contingent display changes in the experiment modulated any foveal load effects. White et al. (2005a) found that preview benefit effects were only modulated by foveal load for participants who did not detect display changes. For those who did detect display changes, no modulatory effect of foveal load was observed, suggesting that there may be individual differences in relation to the nature of parafoveal processing and its modulation by foveal load. More recently, Veldre and Andrews (2018) conducted two experiments to examine foveal load effects. In their first experiment they investigated whether foveal lexical processing difficulty associated with a pretarget word (i.e., manipulated via word frequency) modulated parafoveal semantic processing. They compared fixation times on a target word that had been preceded by an identical preview, a plausible but orthographically unrelated preview, an implausible orthographic neighbor of the target, or an implausible and unrelated preview. Veldre and Andrews failed to find any modulatory effects of foveal load on semantic preprocessing. In their second experiment, they reexamined foveal load effects but used an alternating case preview, a nonword neighbor preview, or a nonword (random consonant-string) preview. The results of this experiment showed an interactive effect between foveal load and parafoveal preview that was limited to effects in the illegal nonword condition. It is noteworthy, though, that this interaction was entirely due to a subset of participants who were aware of display changes that occurred in the experiment. Those participants who were not aware of the changes did not show the effect. Veldre and Andrews argued that their interactive effects were mainly caused by the use of illegal nonword previews as their baseline. It has been demonstrated that participants are more sensitive to changes in boundary paradigm studies when the orthographically illegal nonword previews that are very unlike words are used compared with when wordlike nonword previews are used (Angele, Slattery, & Rayner, 2016); moreover, participants who detected the display changes showed more preview cost, and again it was argued that this was caused by the illegality of the nonword preview (Veldre & Andrews, 2018). Thus, it seems that the interactive effects of the display change awareness with foveal load and preview were very likely due to the salience of an orthographically illegal parafoveal preview that produced disruption to processing of the target word. Kennison and Clifton (1995) also examined individual differences in relation to parafoveal processing and foveal load effects. In their study, they also used the boundary paradigm and assessed preview benefit when the pretarget word was either low-frequency (high foveal load) or high-frequency (low foveal load). Importantly, in line with the suggestion that there exists a relationship between reading ability and working memory capacity as assessed by the Reading Span Test (Daneman & Carpenter, 1980, 1983), Kennison and Clifton (1995) also identified two groups of participants, one with a high reading span and one with a low reading span. Using these participant groups, they tested the hypothesis that high-span readers may show increased parafoveal preview benefits relative to low-span readers, and whether any such effects were modulated by foveal load. The overall pattern of results obtained by Kennison and Clifton did not show the predicted relationship between foveal load and preview benefits. However, when they undertook further analyses to consider reading time data in relation to saccadic launch sites, in the analysis of gaze duration, they did show significant effects consistent with the suggestion that foveal load modulated preview benefit effects. Note, though, that effect held for only 40% of the data and half the participants, suggesting that any effects that did occur were quite specific to a subpopulation. Also, Kennison and Clifton found no evidence to support the suggestion that foveal load and parafoveal processing were themselves influenced by participants’ reading spans. Based on the discussion above, it should be clear that to date there has been only limited work to investigate individual differences in relation to foveal load effects and parafoveal processing in reading. Clearly, further research is required to provide a more comprehensive understanding of the nature of these effects.

Two further studies carried out by Drieghe, Rayner, and Pollatsek (2005), and White (2007) examined the effect of foveal load on word skipping using similar manipulations to those described above. However, in these studies parafoveal words were used that were of different lengths to those used by Henderson and Ferreira (1990). Drieghe et al. (2005) used short target words that were three letters long, and White used short target words that were four letters long. These words were shorter than those used by Henderson and Ferreira (see White et al., 2005a, Footnote 10), and were employed to maximize word skipping. The basic idea in these studies was to investigate whether foveal processing load might affect saccadic targeting as indexed by skipping rates. Both studies failed to demonstrate any interactive effects of foveal load and parafoveal processing in relation to either fixation times on the target word, or skipping rates. Note that this was the case even though skipping rates of the short parafoveal target words was considerable (in Drieghe et al., 2005 it was 37% compared to 16% in White et al., 2005a).

In summary, the evidence for foveal load effects in English reading is mixed with some studies demonstrating such effects and others failing to find evidence for them. It is also true that there is considerable variability in the length of target words for which the boundary paradigm has been used to examine preview benefit in relation to foveal processing load. Finally, it is important to note that the boundary paradigm is most often used to assess the degree, or depth, to which parafoveal information is processed prior to direct fixation rather than the spatial extent of any such processing. Given that mixed results have been obtained across existing studies, it seems valuable to conduct a further, substantive study to investigate foveal load effects in relation to parafoveal processing. However, it would seem sensible to conduct such a study in a language that is visually and linguistically dense, thereby maximizing the possibility that parafoveal processing of text may be operationalized over lexical units (words) that are close to fixation within the perceptual span. It would also be ideal if word length variability in that language was minimal.

Chinese is a visually and linguistically dense language. Also, over 80% of Chinese words are one or two characters long (i.e., word length variability is minimal; Li, Zang, Liversedge, & Pollatsek, 2015; Zang, Fu, Bai, Yan, & Liversedge, 2018; Zang, Liversedge, Bai, & Yan, 2011). For these reasons, in the present experiment we investigated foveal load effects on parafoveal processing in Chinese reading. Additionally, we used single-character target words, thereby ensuring they fell close to fixation within the perceptual span. Furthermore, to ensure statistical power above the recommended value of 0.8 we tested 120 participants based on effect sizes estimated from the previous literature (Veldre & Andrews, 2018).

Before considering our experimental manipulations and hypotheses in further detail, let us next consider the importance of foveal load effects in relation to models of eye movement control in reading. Henderson and Ferreira’s (1990) suggestion that foveal processing load reduces the perceptual span has been incorporated into the basic functional architecture of two very influential such models; the E-Z Reader model and the SWIFT model. Both models have built into their architectures a foveal load mechanism that explains how increased foveal load reduces parafoveal processing. To be specific, the E-Z Reader model, a Sequential Attention Shift (SAS) model, assumes that attention is allocated to one word at a time and word recognition occurs serially and sequentially. Parafoveal processing of the upcoming word begins as soon as attention (but not the point of fixation) shifts to that word. That is, the parafoveal word is processed as soon as attention shifts from the fixated word to the next word, and this happens before the reader’s point of fixation transfers to the upcoming word. Note, however, that when the foveal word is difficult to process, a longer fixation is required to achieve lexical access and the attention shift to the upcoming word is delayed. Accordingly, in such circumstances, this leads to reduced parafoveal preview and less skipping of the upcoming word (e.g., Reichle, 2011; Reichle, Pollatsek, Fisher, & Rayner, 1998). Thus, in the E-Z Reader model a temporally based mechanism is incorporated to produce reduced preview benefits when foveal processing load is high. Note that this mechanism can only produce effects whereby the extent (or depth) to which the parafoveal word is processed is reduced. There is no mechanism in the E-Z Reader model to generate effects consistent with a reduction in the spatial extent of the perceptual span during reading.

In contrast to the E-Z Reader model, the SWIFT model is a model in which processing is guided by attentional gradients (a so-called GAG model) with the central assumption that multiple words within the perceptual span are processed in parallel (Engbert & Kliegl, 2011; Engbert, Nuthmann, Richter, & Kliegl, 2005). As for the effect of foveal processing load on parafoveal processing, the mechanism in SWIFT involves two sources of influence over parafoveal processing, namely the spatial extent and depth of such processing, and these influences are antagonistic in nature. In relation to the spatial dimension, low foveal load causes a wide attentional gradient, while high foveal load leads to a narrow attentional gradient (restricted to the fixated word; Schad, Nuthmann, & Engbert, 2010). That is to say, increased foveal processing difficulty restricts the range of parafoveal words over which processing operates (Engbert & Kliegl, 2011; Kliegl, Nuthmann, & Engbert, 2006; Schad & Engbert, 2012). On the other hand, since the SWIFT model stipulates that words are processed in parallel, then the longer a reader spends fixating a word, the greater the opportunity to process the adjacent parafoveal word. Thus, in relation to the depth to which a parafoveal word is processed, the SWIFT model generates the somewhat surprising prediction that increased foveal processing load (i.e., longer fixations on the foveal word) will result in increased (not decreased) processing of the parafoveal word (Engbert et al., 2005; Kliegl, Hohenstein, Yan, & McDonald, 2013; McDonald, 2005). To be clear, SWIFT stipulates that the depth to which parafoveal words are processed increases as fixation times on the foveal word increase. Empirical evidence in support of this suggestion comes from Schroyens, Vitu, Brysbaert, and d′Ydewalle (1999) and Yan (2015), who showed that larger preview benefits can be associated with high relative to low foveal processing load. Therefore, in the SWIFT model, it seems that there are two antagonistic constraints on parafoveal processing, and a combination of these, potentially, allows for the explanation of null, facilitatory, or inhibitory foveal load effects on parafoveal processing.

In the current experiment, we wished to examine whether foveal processing load at the pretarget word influenced the degree to which a parafoveal target word was processed during Chinese reading. As argued earlier, Chinese is a language with characteristics that are optimal for investigating interactive influences of parafoveal and foveal processing. In our experiment, we created stimuli in which the pretarget foveal word was always a two-character word that was either high or low frequency. In this way, similar to Henderson and Ferreira (1990), we manipulated foveal processing difficulty. Also, in order to ensure that readers’ parafoveal processing of the target word to the right of the pretarget was maximized, and therefore, that the observation of any modulatory influence of foveal processing load on such processing of the parafoveal would also be maximized, target words were always single character words. Thus, (a) through choosing to carry out the experiment in Chinese, which is visually and linguistically dense, and (b) by manipulating foveal load (i.e., word frequency) of pretarget words in a very similar manner to Henderson and Ferreira, and (c) by ensuring that our target words were as short as possible. We assessed the efficacy of parafoveal processing in relation to foveal load through consideration of both target word preview benefit and saccadic targeting. Consideration of preview benefit and skipping allows us to determine whether foveal load effects impact decisions of where and when to move the eyes comparably. In line with the foveal load hypothesis, we predicted that if foveal processing load reduces the depth or extent to which a parafoveal word is processed, then we should obtain interactive effects of pretarget word frequency and target word preview, such that the preview benefit on the target word would be reduced, and target word skipping would be reduced when the pretarget word is low-frequency relative to when it is high-frequency.

Beyond our investigation of foveal effects on parafoveal processing, our experiment provides an opportunity to examine a more controversial, theoretical issue—that is parafoveal-on-foveal (PoF) effects. PoF effects occur when the lexical characteristics of a yet-to-be-fixated parafoveal word influence fixation durations on the currently fixated foveal word. A recent, very compelling study by Brothers, Hoversten, and Traxler (2017) examined whether the lexical frequency (high vs. low) of a parafoveal word had any influence on fixation times on the foveal word. One reason why this study was so impressive was that a very large number of participants (244)- was tested. A large number of participants such as this ensures against suggestions that any lack of lexical PoF effects might have arisen due to a lack of experimental power. It is noteworthy that even with such a large number of participants, Brothers et al. found no evidence of lexical PoF effects. In their study, Brothers et al. manipulated a lexical characteristic of parafoveal words, namely, their frequency. Our experiment also involves the manipulation of a lexical characteristic of the parafoveal word, namely, the target word’s lexical status. Recall that in the present experiment we will orthogonally manipulate pretarget word frequency with target word preview. This means that in two of our experimental conditions, when participants fixate the pretarget word the parafoveal character will be a word, and in the other two conditions it will be a pseudocharacter. Thus, if participants are sensitive to the lexical status of a parafoveal character prior to its direct fixation, as parallel models of eye movement control (e.g., SWIFT) stipulate, then it seems reasonable to suggest that PoF effects should be observed. Thus, if PoF effects occurred, as per the predictions of SWIFT, then we should see increased fixation times on the pretarget word when the target was a pseudocharacter compared to when it was a word. The alternative view, that words are processed serially and sequentially (e.g., as per the E-Z Reader model), predicts that no PoF effects should occur since the lexical processing of parafoveal words should not begin until lexical foveal processing is completed. Once again, we draw the reader’s attention to the fact that the present experimental conditions (described in detail above) are optimal for the observation of such effects.

## Method

### Participants

One hundred and 20 students (Mean Age = 21.8 years, *SD* = 1.8; Male = 18, Female = 102) from Tianjin Normal University took part in the experiment. The participants were all native Chinese speakers with normal or corrected-to-normal vision and no history of reading impairments. They were naïve with respect to the purpose of the experiment. All of them received a gift for their participation.

### Apparatus

Movements of readers’ right eyes were monitored by an SR Research Eyelink 1000 system at a sampling rate of 1,000 H_Z_. Due to experimental testing constraints, we were forced to run this experiment during two different sessions using a different type of display monitor in each (a 17-in. SAMSUNG SyncMaster 959NF monitor with a 1024 × 768 pixel resolution and a refresh rate of 120 H_Z_; a 19-in. DELL monitor with a 1024 × 768 pixel resolution and a refresh rate of 150 H_Z_). To assess any influence of the type of presentation screen we included this variable in our analyses (and to preempt our results, there were no significant effects caused by the two different display monitors in any of our analyses). The viewing distance between the participant and the screen was 61 cm. Stimuli were presented in black in Song font with a white background. One Chinese character subtended about 1.0° of visual angle.

### Materials and Design

We manipulated foveal processing load by making the pretarget word either high- or low-frequency. To do this, 64 two-character verbs were selected from Cai and Brysbaert (2010), of which 32 were high-frequent and the other 32 were low-frequent. Each high-frequency word was paired with a low-frequency word. High-frequency words (53.7–1,521.7 occurrences per million) were significantly more frequent than low-frequency words (0.03–0.92 occurrences per million), *F*(1, 31) = 21.49, *p* < .001, but the words did not differ in their stroke complexities (14–20 strokes; *F*(1, 31) = 0.15, *p* = .704). The means and standard deviations for each condition are shown in Table 1.[Table-anchor tbl1]

Thirty-two single-character nouns were selected as the target words. The target words had frequencies ranging between 10.3–47.1 occurrences per million (*M* = 24.4, *SD* = 10.6) and between 8–15 strokes (*M* = 10.3, *SD* = 1.9). The preview of the target word was manipulated by using the boundary paradigm such that the participant had either an identical preview (i.e., the target itself) or a pseudocharacter preview. We created 32 pseudocharacters by using the Private Character Editor in Windows 10 system. To be specific, we selected radicals from different real characters and then positioned the radicals legally (i.e., the positions of the radicals may appear in real characters) to create a pseudocharacter; that is, the novel combination of the radicals followed the orthographic structure of a Chinese character and the pseudocharacters were orthographically legal. Moreover, the pseudocharacter preview did not share any semantically, phonetically, or visually similar radicals with its corresponding target word, but was matched to the target word in relation to its stroke number (*M* = 10.3, *SD* = 1.8), *F*(1, 31) = 0.33, *p* = .572. The experiment was a 2 (Foveal Load: Low, High) × 2 (Parafoveal Preview: Identical, Pseudocharacter) within subject design, with 32 sentences for each condition.

**Figure 1 fig1:**
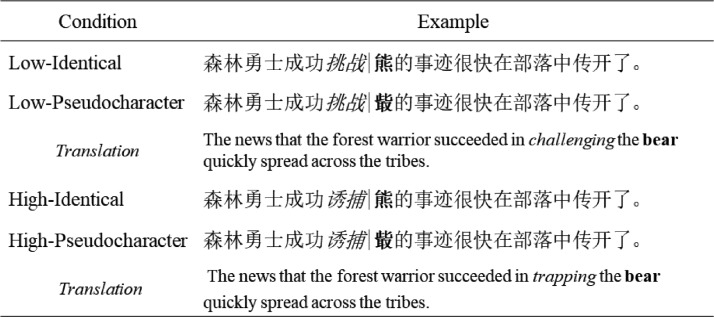
An example set of sentences under the four conditions. The pretarget words are presented in italics while the previews of the target are in bold (for illustration purposes only). The vertical black line represents the position of the invisible boundary. As readers’ eyes crossed the boundary, the preview was replaced by the target.

The pretarget word and the target word were then embedded into sentences. For each pretarget word pair (i.e., the high- and low-frequency counterparts), the same target word followed. The target word fitted well within the sentence frame and sentential content up to the pretarget word was identical across conditions. Around 58% of the pretarget and target pairs comprised verb–object combinations in the sentences. In all, 32 sets of sentence frames were created. All the sentences were between 16 and 22 characters in length (*M* = 20 characters). The naturalness of all sentences and the predictability of both the pretarget and the target words were rated by three different groups of participants from Tianjin Normal University who did not take part in the eye tracking study. Thirty-four participants were asked to evaluate the naturalness of the sentences on a 5-point scale (1 = *very unnatural*, 5 = *very natural*), with 17 in each foveal load condition. The mean naturalness score was 3.9 (*SD* = 0.3) with no difference between low- and high-load conditions, *F*(1, 31) = 0.28, *p* = .602. To assess the predictability of the pretarget words, 18 different participants were required to carry out a cloze task and provide the word following the text up to the position of the pretarget word. A further 36 participants rated the predictability of the target word (18 in each different foveal load condition) by performing the cloze task for the sentence content up to the target word. The mean predictabilities of the pretarget and the target word were very low (see Table 1) without any significant differences between low- and high-load conditions (for pretarget words, *F*(1, 31) = 0.91, *p* = .347; for target words, *F*(1, 31) = 2.65, *p* = .113). The boundary paradigm was used to present an identical or a pseudocharacter preview of the target, in which the preview would be replaced by the target word when the reader’s point of fixation crossed the invisible boundary located before the target position. An example set of the sentences is shown in Figure 1.[Fig-anchor fig1]

Four files were constructed. Counterpart sentences from each set of four were allocated to one of the four files according to their experimental condition. Experimental conditions were rotated across files according to a Latin square. Each file included 32 experimental sentences of which eight sentences were from each of the four conditions. All participants saw all of the 32 experimental sentences in a file, and each participant saw each sentence only once. Additionally, the same 24 filler sentences were included in each file. A total of 46% experimental and filler sentences were followed by a yes/no comprehension question that participants were required to answer by pressing a “Yes” or “No” button. All the sentences were presented in a random order. In addition to the experimental and filler sentences, six practice sentences, four of which were followed by a comprehension question, were presented at the beginning of each testing session.

### Procedure

Participants were tested individually. After arriving for the experiment, participants were instructed that they would read some sentences and should understand them to the best of their ability and then press a button to terminate the trial. They were informed that there would be a comprehension question after some of the sentences they would read. Answers by pressing response buttons to questions were recorded. Before recording the participants’ eye movement data, a 3-point horizontal calibration procedure was used. We ensured an average calibration error below 0.35 degrees. During testing, each trial began with a drift check at the beginning of the sentence. The participant was recalibrated if the value of the drift correct was greater than 0.35 degrees. The experiment lasted approximately 25 min.

### Power Analysis

A meta-analysis reported in Veldre and Andrews (2018) has shown an average effect size of 0.09 (Cohen’s *d*) for the interaction of foveal lexical processing load and parafoveal preview, calculated from means and variance estimates across studies. However, for the studies of Henderson and Ferreira (1990) and White et al. (2005a) that have reported very robust modulatory effects of foveal load on preview benefit, the effect sizes were 0.52 and 0.43, respectively. Given that the experimental design and the nature of the manipulations in the present study were directly comparable to the design (2 × 2 within subjects design) and manipulations (foveal load: high- vs. low-frequency and Preview: identity vs. random letter string) adopted by White et al., we adopted 0.43 as a prior effect size. A power analysis was then conducted using the software developed by Westfall (2015). This analysis indicated that at least 20 participants per condition are required for 32 stimuli to achieve a power of 0.8, the minimum power value recommended by Cohen (1962). Therefore, our sample size of 30 participants and 32 sentences per condition (i.e., 120 participants and 128 stimuli in total) meant that the power in the present study exceeded the minimum required, and indeed as we intended, allowed us to carry out a strong test of the foveal load hypothesis.

## Results

The overall comprehension accuracy was 91% indicating that participants read the sentences properly and understood them well. Before analyzing the data, we removed any fixations shorter than 80 ms or longer than 1,200 ms. Trials were excluded in which (a) a track loss occurred or there were fewer than five fixations in total (0.26% of the data); (b) participants blinked while the display changed or while they were fixated on the target word, as well as trials in which the display change occurred early or was delayed (for the target word analyses: 15.02%); (c) any observations for each fixation time measure and each participant were more than 3 standard deviations from that participant’s mean (for the target word analyses: 0.17%; for the pretarget word analyses: 1.13%).^1^

We examined fixation times and skipping probability for the target, as well as for the pretarget word. To be specific, we considered the following measures: skipping probability (SP; the probability that readers skip a word during first pass reading), first fixation duration (FFD; the duration of the first fixation on a word during first pass reading), single fixation duration (SFD; the duration of a fixation on a word when it is the only fixation made on that word during first pass reading), and gaze duration (GD; the sum of all fixations on a word before making a saccade to another word during first pass reading). The means and standard deviations for these measures are shown in Table 2.[Table-anchor tbl2]

To analyze the data, we constructed Linear Mixed Models (LMMs) by using the lme4 package (Bates, Mächler, Bolker, & Walker, 2015) in R 3.4.4 (R Core Team, 2018) and RStudio (2018). Fixation durations were log-transformed prior to running the models. For skipping probability, logistic LMMs were used. Foveal processing load and preview were treated as fixed factors and were contrasted by using the function of “contr.sdif()” in MASS package. Participants and items were specified as crossed random effects, with both random intercepts and random slopes (Barr, Levy, Scheepers, & Tily, 2013). When we ran the models we always began with full models that included the maximum random effects structure. But the slopes were removed if the model failed to converge (indicating overparametrization). We calculated *p* values based on Satterthwaite’s approximations using the lmerTestpackage (Kuznetsova, Brockhoff, & Christensen, 2017).

### The Pretarget Word

Fixed effect estimations for the eye movement measures at the pretarget word are reported in Table 3. Consistent with numerous previous studies (e.g., Juhasz & Pollatsek, 2011; Kliegl, Grabner, Rolfs, & Engbert, 2004; Liversedge et al., 2014; Rayner & Duffy, 1986; see also Rayner, 1998, 2009), frequency effects on the pretarget word occurred for both fixation times and skipping probability. Low frequency pretarget words caused longer fixations and reduced skipping rates than high frequency pretarget words (Fixation times: all *t*s > 4.16, *p*s < .001; SP: *z* = −3.57, *p* < .001). Clearly, our manipulation of foveal processing load was highly effective. In addition, at the pretarget word, there were no effects of parafoveal preview, nor foveal load and preview show any interactive effects across all measures (Fixation times: all |*t*|s < 0.69, *p*s > .05; SP: |*z|*s < 1.18, *p*s > .05). There was no hint of any parafoveal-on-foveal lexical status effect. This finding is in line with the results of Brothers et al. (2017), providing no evidence for parallel lexical identification of words during Chinese reading.[Table-anchor tbl3]

### The Target Word

Fixed effect estimations for the eye movement measures at the target word are presented in Table 4. For skipping probability, we found that readers skipped the target word less when the pretarget word was difficult relative to when it was easy (*z* = −2.72, *p* = .007). Furthermore, Chinese readers were much less likely to skip the target word when they had a pseudocharacter preview relative to an identity preview (*z* = −7.52, *p* < .001). However, very importantly in relation to our primary theoretical question, foveal load and preview did not interact (*z* = −0.37, *p* = .711), in other words, the two factors affected skipping independently. For fixation times on the target word, there was no reliable difference between the low- and high-foveal processing load conditions indicating that foveal processing load did not spillover from the pretarget word to the upcoming target word (all |*t|*s < 1.04, *p*s > .05). As expected, all the fixation time measures showed very significant preview benefit effects, with pseudocharacter previews leading to longer reading times in relation to identical previews (all *t*s > 5.19, *p*s < .001). Similar to word skipping, foveal load and parafoveal preview did not interact for the fixation time measures, that is, foveal load did not modulate parafoveal processing (all |*t|*s < 0.91, *p*s > .05). These findings are very similar to those reported in previous studies by such as Drieghe et al. (2005), Veldre and Andrews (2018), White (2007), and Liu, Reichle, and Li (2015; also see Marx, Hawelka, Schuster, & Hutzler, 2017; Vasilev, Slattery, Kirkby, & Angele, 2018, in Exp. 1 and 2b); and are inconsistent with the findings reported by such as Henderson and Ferreira (1990), White et al. (2005a), and Yan (2015); also see Angele et al., 2016; Luke, 2018; Vasilev et al., 2018, in Exp. 2a).[Table-anchor tbl4]

### Additional Analyses

**Figure 2 fig2:**
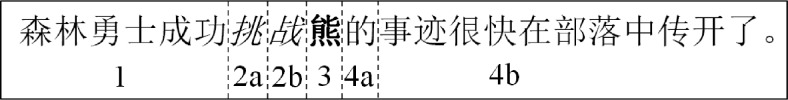
Regions of interest in the example sentence. Region 1 is the region prior to the pretarget word. Region 2a is the first character of the pretarget word, and Region 2b is the second character of the pretarget word. Region 3 is the single character target word. Region 4a is the first character after the target word and Region 4b is the remainder of the sentence.

Consider Table 5, which provides an illustration of the probability of saccade launch and corresponding landing sites for every saccade that crossed the invisible boundary during first pass reading. These data are collapsed across the experimental conditions of our experiment. We have quantified these probabilities in relation to six regions (see Figure 2) - the region prior to the pretarget word, the first character of the pretarget word, the second character of the pretarget word, the target word itself, the first character after the target word, and the remainder of the sentence.[Table-anchor tbl5][Fig-anchor fig2]

These data provide descriptive details of the nature of first pass saccades and fixations around and on the target word. Several aspects of Table 5 are noteworthy. First, while the target word was fixated on the majority of occasions, it was also skipped quite frequently (42% of trials). Focusing on the trials where the target word was fixated, we can see that saccades landing on the target were most often launched from the preceding word (82%), with only a minority of saccades landing on the target when they were launched from further than a word away (18%). In relation to the trials where the target word was skipped, we can also see that such saccades were launched from the pretarget word on the vast majority of occasions (87%). Furthermore, on the majority of occasions that the target was skipped, the fixation landed on the posttarget character (72%). Only rarely did readers skip the target word with a saccade launched from before the pretarget word to land at a point beyond the posttarget character. These results are entirely consistent with the established finding that the perceptual span in Chinese is 2–3 characters to the right of fixation (Chen & Tang, 1998; Inhoff & Liu, 1998).

As already noted, the target in the present study was a single-character word that was often skipped. Potentially, the diminished data set might have contributed to our failure to obtain evidence for modulatory influences of foveal load on preview benefit as shown on fixation time measures. In order to ensure that this was not the case, we created an additional region of analysis that combined the target word and the post target character (the post target character was a single character word for 84% of the stimuli. Of these 69% were function words). In this analysis, the probability that the region was fixated was 88%. Further analyses of fixation time data (see Table 2 for means and standard deviations) from the combined region including the target word and the posttarget character, again, showed no modulatory effect of foveal load on preview benefit (see Table 6, all |*t*|s < 1.10, *p*s > .05), reinforcing our claim that foveal load did not modulate parafoveal processing.[Table-anchor tbl6]

Note also that our use of a two character pretarget word was effective in ensuring that this attracted a pretarget fixation (85% of trials), thereby ensuring that on most of the trials the target word fell in parafovea prior to a saccade that transgressed the boundary. Of these occasions, note also that readers made more saccades from the second character of the pretarget word (62%) than from the first character of the pretarget word (38%). Finally, recall that 76% of fixation occurrences on pretarget words were single fixations, suggesting that Chinese readers were less likely to make a refixation on a word when their initial fixation was at the end compared with the beginning of a word consistent with the previous findings (e.g., Yan, Kliegl, Richter, Nuthmann, & Shu, 2010; Zang, Liang, Bai, Yan, & Liversedge, 2013).

Next, let us consider the forward saccade length data for all fixations launched from the pretarget word. On average, when readers made a saccade from the pretarget word, those saccades were approximately two characters long (*M* = 1.94 Characters, *SD* = 0.83). Analyzing these data in relation to our experimental variables, we found that, there was a reliable main effect of foveal load (*b* = −0.08, *SE* = 0.02, *t* = −4.37, *p* < .001), and a reliable main effect of parafoveal preview (*b* = −0.14, *SE* = 0.02, *t* = −7.63, *p* < .001) but no significant interaction between the two factors (*b* = −0.03, *SE* = 0.02, *t* = −1.14, *p* = .256). To be specific, when foveal processing load was high readers made shorter saccades (*M* = 1.93 characters) than when foveal processing load was low (*M* = 2.08 characters). And readers made shorter saccades when the parafoveal preview was a pseudocharacter (*M* = 1.87 characters) than when it was an identity preview (*M* = 2.14 characters). Unsurprisingly, this finding is entirely consistent with the target word skipping results reported earlier. Taken together, the skipping and saccade length effects indicate that foveal lexical processing load and parafoveal preview validity independently influenced saccade targeting in Chinese reading, consistent with findings from English reading reported by Drieghe et al. (2005). However, the present findings are inconsistent with those of a Chinese reading study conducted by Liu et al. (2015). They used high- or low-frequency foveal words with a valid or an invalid parafoveal preview and found a foveal load effect on saccade length that was modulated by parafoveal preview. That is, high foveal processing load caused a shorter forward saccade than low processing load but only when the preview was valid. It should be noted that Liu et al. manipulated the invalid-preview by replacing the entirety of the remainder of the sentence after the boundary with ※ symbols. Previews such as this are quite different in their visual appearance relative to a pseudocharacter preview. Furthermore, the spatial extent of the preview was significant. It seems likely, therefore, that there would be little difference in the degree to which readers were able to process the symbol preview regardless of foveal load. In contrast, under identity conditions, differential parafoveal processing could readily occur, and in such circumstances, interactive effects would occur. To be clear, while at first glance it may appear that there are inconsistencies between the results of the current study and those of Liu et al., upon closer examination of the precise conditions under which the effects occurred, it appears that the results may, in fact, be compatible.

### Bayesian Analyses

In order to provide further statistical support for the null interaction of foveal load and parafoveal preview, we undertook Bayes factor analyses for linear mixed models (Morey et al., 2018) in relation to first fixation duration, single fixation duration, and gaze duration. Bayes factors both for the full model (i.e., *BF*_Full_, the model containing the main effects of foveal load and parafoveal preview, and their interaction) and the model with only main effects (i.e., *BF*_Main_) were calculated. By comparing the two models (*BF* = *BF*_Full_/*BF*_Main_), we were able to evaluate the nonsignificant interaction between foveal load and preview. *BF* values smaller than 1 favor the null hypothesis, whereas *BF* values greater than 1 favor the alternative hypothesis. For each of the reading time measures we used the default scale prior (*r* = .5) and 100,000 Monte Carlo iterations of the BayesFactor package. The results of Bayesian analysis favored the null hypothesis (First Fixation Duration: *BF* = 0.10, Single Fixation Duration: *BF* = 0.08, Gaze Duration: *BF* = 0.08). Also, a sensitivity analysis with different priors (i.e., .2, .3, .4, .5, .6, .7, and .8) provided consistent results (all *BF*s < 0.30).

## Discussion

The current study was aimed at investigating foveal load effects on parafoveal preprocessing. To be specific, we examined modulatory effects of processing difficulty of a two-character pretarget word for preview benefit at a subsequent one-character target word, and for saccadic targeting in relation to that word during normal Chinese reading. The analysis of the pretarget word showed robust word frequency effects for fixation times (FFD: 14 ms, SFD: 15 ms, GD: 45 ms), indicating an effective manipulation of foveal lexical processing load. Also, on more than 80% of occasions when readers fixated the target word, or when they skipped it, they launched their saccade from the preceding word, suggesting that our use of a two-character pretarget word was effective in causing readers to fixate it prior making a saccade to cross the boundary. Thus, as was our intention, prior to the boundary change, we ensured that the target word fell as close to fixation as possible. This maximized the depth to which the target character was processed before it was fixated or skipped. In short, the design characteristics of our experiment appear to have worked effectively and in exactly the manner we had hoped in relation to the experiment providing an effective test of our hypotheses.

According to the Foveal Load Hypothesis advocated by Henderson and Ferreira (1990), it was predicted that parafoveal preview benefit of the target word as well as the skipping probability of the target word would be reduced when pretarget word processing was difficult relative to when pretarget word processing was easy. Given that our target words had minimal horizontal spatial extent and were positioned close to fixation prior to the boundary change, we felt that we had optimized experimental conditions under which we might observe the influence of foveal processing load on the depth to which the upcoming target word was processed.

Although we did observe very reliable main effects of preview benefit at the target word, counter to our predictions, we obtained absolutely no evidence of a modulatory effect of foveal load on the preview benefit. We conclude, therefore, that foveal processing load does not modulate the (linguistic) depth to which an upcoming single character word is processed during normal Chinese reading. By this we mean that foveal load did not influence how effectively an upcoming single character Chinese word was linguistically processed prior to it being directly fixated or skipped. We note that similar effects have been reported for some studies conducted in alphabetic language reading (e.g., Drieghe et al., 2005; Drieghe, Fitzsimmons, & Liversedge, 2017; Marx et al., 2017; Vasilev et al., 2018; Veldre & Andrews, 2018; White, 2007). And we note also that in these studies words with a greater horizontal spatial extent were employed compared with the words used in the present experiment (due to the stimuli being comprised of multiletter alphabetic words). As we noted earlier, there was considerable variance of the target word length across previous studies, and word length has been shown to strongly affect the degree to which readers effectively process parafoveal previews (e.g., Juhasz, White, Liversedge, & Rayner, 2008; White, Rayner, & Liversedge, 2005b). It is possible, therefore, that target word length differences in previous studies contributed to the different patterns of effects that were obtained (e.g., Drieghe et al., 2005, 2017; Kennison & Clifton, 1995; Marx et al., 2017; Vasilev et al., 2018; Veldre & Andrews, 2018; White, 2007). On the basis of the present results, and also in the context of the alphabetic studies mentioned earlier, it seems reasonable to conclude that the present failure to obtain foveal load effects on parafoveal processing could not be due to our target words projecting horizontally away from fixation to a point beyond the range over which such processing operated. Clearly, our single-character Chinese words had minimal horizontal spatial extent, and therefore, it is almost certain that they were available in the parafovea to be processed prior to a saccade across the boundary. Recall that here we have distinguished between two possible types of parafoveal processing in relation to foveal load; the linguistic depth to which a parafoveal word might be processed and the spatial extent of parafoveal processing. With respect to linguistic depth, here we are referring to the extent to which a parafoveal word may be, potentially at least, processed orthographically, phonologically, morphologically, syntactically, and/or semantically before it is directly fixated. The present results provide strong evidence that foveal load does not modulate the depth to which a very short parafoveal word is linguistically processed prior to a saccade to or beyond it. If such effects had occurred, then we would have expected that our target words would have been processed visually and linguistically to a greater depth when the pretarget word was high-frequency compared with low-frequency. However, to reiterate, preview benefits were very similar for both types of pretarget words with no modulation by foveal load.

The second way in which modulatory foveal load effects might impact parafoveal processing is through a reduction in the horizontal spatial extent of such processing. That is, under high foveal processing load conditions, the horizontal rightward window over which parafoveal processing operates may be reduced or truncated. Essentially, this amounts to a reduction in the perceptual span in reading under high foveal load conditions. A study that is very relevant to this idea was reported by Yan, Kliegl, Shu, Pan, and Zhou (2010), who examined how processing difficulty (manipulated by word frequency) of word N + 1 in the parafovea modulates preview of word N + 2 within the perceptual span. Yan et al. showed that preview benefit for an identical compared with an unrelated word preview for word N + 2 only occurred when word N + 1 was high frequency and easy to process. While the present study offers a strong test of the depth of the parafoveal processing hypothesis, it is a much less stringent test of the processing extent hypothesis. Yan et al.’s study, however, potentially supports this alternative account of foveal load effects, that is, the truncation of the spatial extent of parafoveal processing. They showed the spatial extent of parafoveal processing was reduced with an increment of parafoveal processing load (though, note, there was no manipulation of foveal processing load in this study). Indeed, to our knowledge, there has been no study to date that has directly investigated whether incrementally increasing foveal load systematically decrements the spatial extent of parafoveal processing in reading. This is clearly an issue for future research.

A further issue concerns how the present results relate to existing results from investigations of foveal load effects in Chinese reading. In this respect, arguably the most relevant study to the present study is that of Yan (2015). Yan manipulated foveal load at a pretarget word using more or less visually complex characters (a manipulation of the number of strokes) alongside a manipulation of the preview of a two-character target word (either an identity preview or a preview formed from two Chinese characters that together did not form a word) using the boundary paradigm. Yan reported effects indicating a “reversed” influence of foveal load relative to effects more widely reported. That is, Yan found that increased foveal visual processing load produced increased, not decreased, preview benefit. Among studies that have actively manipulated foveal load in relation to preview of a target word, this result sits in isolation. All the existing studies that have shown modulatory influences of foveal load on parafoveal processing have shown an increased cost under high than low load conditions. Furthermore, the remaining studies that did not show such modulatory effects actually failed to show any relationship between foveal load and parafoveal processing. Among studies that directly manipulated foveal load and previews, the study by Yan is the only one in which reversed foveal load effects have been reported and at present, to us, the reasons for the different pattern of effects in Yan’s study are not entirely clear. One point that we do note, though, is that Yan did not report the predictabilities of parafoveal words in the two different foveal load conditions. Predictability of words has been demonstrated to strongly influence parafoveal preview (e.g., Balota, Pollatsek, & Rayner, 1985; White et al., 2005b) and, therefore, if differences in this variable did exist across conditions, this could potentially have affected the results.

Up to now, our discussion has focused on how foveal load failed to modulate parafoveal processing as indexed by preview benefit; that is, we assessed the influence of foveal load on decisions of when to move the eyes from the target word. However, in our experiment we also investigated how foveal load influenced decisions about where to move the eyes; that is, how it affected saccadic targeting decisions. The present results clearly showed that processing difficulty associated with the pretarget word and the preview validity of the target word independently affected the decision of whether or not to skip the target word, as well as where to target the next forward saccade. We found that readers skipped the target word more often under low than high foveal load conditions. We also found that readers made longer saccades from the pretarget word under low than high foveal load conditions. In order to gain greater clarity over these two effects, we undertook an additional analysis to explore whether the forward saccade length effect from the pretarget word might be exclusively driven by skips of the target word. To do this, we examined the mean saccade length from the pretarget word for only those trials in which the target word was not skipped. Under this analysis there were reliable main effects of foveal load and parafoveal preview, and no interactive effect between the two factors (Foveal load effect: *b* = −0.10, *SE* = 0.02, *t* = −4.59; Parafoveal preview effect: *b* = −0.10, *SE* = 0.02, *t* = −4.84; Foveal Load × Parafoveal Preview: *b* = −0.01, *SE* = 0.03, *t* = −0.34). When the pretarget region was difficult to process, readers made shorter saccades to the target word (*M* = 1.56 characters) than when the pretarget word processing was easy (*M* = 1.72 characters). And readers were more likely to make shorter saccades to the target word when the parafoveal preview was a pseudocharacter (*M* = 1.56 characters) than when it was an identity preview (*M* = 1.72 characters). Both these results suggest that saccadic targeting behavior was affected generally, with increased difficulty causing both reduced skipping and shorter saccades from a word, both of which are in line with the claim advocated by Drieghe (2008), that readers tend to adopt a more conservative strategy in relation to processing the upcoming words in a sentence when the currently fixated word is difficult to process; by contrast, when the current word is easy to process, it appears that readers adopt a more relaxed strategy, engaging with upcoming material to an increased degree, and therefore, targeting their saccades further into the upcoming text. Thus, foveal load influences saccadic targeting both in relation to the distance readers move their eyes into the text, and the extent to which they skip upcoming words in the text.

A final aspect of our results that is noteworthy concerns the lack of frequency spillover effects from the pretarget word at the target word. Although a number of experimental studies have shown word frequency spillover effects (e.g., Angele et al., 2016; Drieghe et al., 2005; Kennison & Clifton, 1995; Luke, 2018; Rayner & Duffy, 1986; Rayner, Sereno, Morris, Schmauder, & Clifton, 1989; Veldre & Andrews, 2018; White, 2008), it should be noted that when such effects have occurred, they are often relatively small (e.g., Drieghe, Rayner, & Pollatsek, 2008; Pollatsek, Juhasz, Reichle, Machacek, & Rayner, 2008). Furthermore, there are several other studies that have failed to find such effects (e.g., Liu et al., 2015; White et al., 2005a; Vasilev et al., 2018). Note that, it has been argued that a spillover effect may be a consequence of reduced parafoveal preview (Pollatsek et al., 2008; Rayner & Duffy, 1986; Rayner et al., 1989; Reichle, 2011), and in this respect, our finding of no spillover effect in relation to the pretarget word may be interpreted as a failure to observe a reduced preview of the target word. If this interpretation is correct, then it is entirely consistent with what we argued earlier. What is very clear, however, is that currently, we remain uncertain as to the circumstances under which frequency spillover effects do, and do not, occur. This is a further issue that future research must address.

Next, we will briefly consider the current findings in relation to models of eye movement control in reading. First, let us consider the E-Z Reader model and its specification of the relationship between foveal load, parafoveal processing, and word skipping. According to the E-Z Reader model foveal load affects the amount of parafoveal preview and whether or not an upcoming word is skipped (see Drieghe et al., 2005; Reichle & Drieghe, 2013). When foveal processing is difficult, a reduced parafoveal preview of the following word leads to a reduced skipping probability of that word. Conversely, when the foveal word is easy to process, there is a greater preview of the upcoming word, resulting in an increased probability that it will be skipped. Thus, to some extent, there is a discrepancy between the current findings and the specifications of E-Z Reader in relation to foveal load effects on preview and word skipping. Our results suggest that foveal load did influence word skipping directly, but not via a reduction in preview of the subsequent word. That is to say, the current findings indicate that the mechanism associated with foveal load and that associated with decisions of whether or not to skip the next word should be decoupled within the computational architecture of E-Z Reader (see also Drieghe et al., 2005 for a similar conclusion). On the other hand, when we look back to the predecessor of the E-Z reader model, that is, Morrison’s (1984) model, the computational specification seems to predict a null foveal load effect with respect to both depth and extent of parafoveal processing. Morrison assumed that lexical access of the currently fixated word causes both an attention shift and saccadic program to the next word. Furthermore, Morrison’s model assumes that processing of parafoveal information is constant and, therefore, uninfluenced by foveal processing load. Accordingly, foveal load should not directly modulate the depth, nor the extent of parafoveal processing—clearly, this fits with the present findings. We fully acknowledge that limitations have been identified with Morrison’s model (e.g., see Rayner, 1998; Reichle et al., 1998). However, our intention in considering it in relation to our findings is simply to demonstrate that there is an existing computational architecture that can account for our effects.

Next, let us consider our findings in relation to the SWIFT model. In the SWIFT model, the degree to which an upcoming word is processed in the parafovea is determined by the duration of the fixation on the word preceding it. Longer fixations result in increased preview, while shorter fixations result in decreased preview. Thus, as specified earlier, according to SWIFT, increased foveal processing load that causes longer fixations will result in greater preview of the upcoming word. Clearly, we found no evidence to support this suggestion. Furthermore, we also failed to observe any effects of the lexical status of the target preview at the pretarget word; that is, a parafoveal-on-foveal effect. To the extent that SWIFT specifies that words are lexically processed in parallel, our result is also inconsistent with its specifications. This is particularly so given that Chinese is an unspaced, dense language (Liversedge et al., 2016), and we utilized single-character target words that were directly adjacent to the pretarget region (thereby maximizing the possibility that parallel lexical processing effects might be observed). More generally, the present results are consistent with the conclusions of Brothers et al. (2017). It should be clear that current implementations of computational models of eye movement control (and here we consider that Morrison’s model is not currently implemented) are unable to fully account for the present findings.

In conclusion, the present study provides a robust test of one version of the Foveal Processing Load Hypothesis, examining whether the difficulty of a pretarget word influences the degree to which an upcoming short target word is processed. We examined Chinese reading and used single-character target words to maximize the possibility of parafoveal processing of the target word. Even though our manipulation of foveal processing load was very effective, we obtained no evidence of any modulatory influence of such load on the depth to which a one-character upcoming word is processed during natural Chinese reading. However, our results clearly showed that saccadic targeting, in relation to forward saccade length from the pretarget word and in relation to target word skipping, is influenced by foveal load and this influence occurs independent of parafoveal preview.

## Figures and Tables

**Table 1 tbl1:** The Mean Statistical Characteristics of the Pretarget Word and the Experimental Sentence Under Low and High Foveal Load Conditions (SDs in Parentheses)

Condition	Frequency (*per million*)	Stroke number	Sentence naturalness	Predictability of the pretarget word (*%*)	Predictability of the target word (%)
Low foveal load	242.9 (295.7)	16.8 (1.8)	4.0 (0.3)	1.3 (3.7)	2.3 (4.9)
High foveal load	0.4 (0.3)	16.6 (1.9)	3.9 (0.3)	.6 (2.5)	4.5 (7.7)

**Table 2 tbl2:** Eye Movement Measures for the Pretarget Word, the Target Word, and the Combined Target Word and Posttarget Character Across Conditions (SDs in Parentheses)

Measure	Number of observations (included in the analysis/all)	Low-identical	Low-pseudocharacter	High-identical	High-pseudocharacter
Pretarget					
Skipping probability	3830/3840	.18 (.19)	.16 (.18)	.13 (.15)	.12 (.15)
First fixation duration (ms)	3217/3263	238 (44)	236 (40)	251 (45)	252 (51)
Single fixation duration (ms)	2463/2491	238 (47)	236 (44)	255 (55)	252 (59)
Gaze duration (ms)	3207/3263	283 (78)	288 (81)	328 (106)	333 (115)
Target					
Skipping probability	3253/3840	.50 (.25)	.37 (.24)	.46 (.24)	.33 (.25)
First fixation duration (ms)	1877/2345	257 (62)	286 (72)	257 (67)	291 (73)
Single fixation duration (ms)	1741/2158	258 (61)	297 (84)	257 (68)	296 (75)
Gaze duration (ms)	1875/2345	261 (63)	309 (79)	267 (76)	316 (90)
The combined region of the target and posttarget character					
First fixation duration (ms)	2848/3332	254 (46)	277 (63)	248 (49)	276 (60)
Single fixation duration (ms)	2068/2492	257 (50)	295 (81)	249 (55)	291 (70)
Gaze duration (ms)	2836/3332	309 (79)	366 (99)	308 (88)	360 (95)

**Table 3 tbl3:** Fixed Effects Estimates From the (Generalized) Linear Mixed-Effects Models and 95% Confidence Intervals for the Eye Movement Measures at the Pretarget Word

Fixed effect	*b*	CI	*SE*	*t/z*	*p*
Skipping probability^a^					
Foveal load (Low vs. High)	**.39**	**[.33, .45]**	**.12**	**−3.57**	**<.001**
Preview (Identical vs. Pseudocha.)	.47	[.42, .52]	.10	−1.17	.242
Foveal load × Preview	.51	[.41, .60]	.19	.16	.874
Display monitor (First vs. Second)	.52	[.41, .64]	.24	.41	.680
First fixation duration					
Foveal load (Low vs. High)	**.05**	**[.03, .07]**	**.01**	**4.59**	**<.001**
Preview (Identical vs. Pseudocha.)	−.00	[−.02, .02]	.01	−.22	.828
Foveal load × Preview	.00	[−.04, .05]	.02	.10	.921
Display monitor (First vs. Second)	−.05	[−.10, .00]	.03	−1.78	.077
Single fixation duration					
Foveal load (Low vs. High)	**.05**	**[.03, .08]**	**.01**	**4.17**	**<.001**
Preview (Identical vs. Pseudocha.)	−.01	[−.03, .02]	.01	−.52	.605
Foveal load × Preview	−.00	[−.05, .05]	.03	−.00	.999
Display monitor (First vs. Second)	−.05	[−.10, .01]	.03	−1.68	.096
Gaze duration					
Foveal load (Low vs. High)	**.12**	**[.08, .15]**	**.02**	**6.95**	**<.001**
Preview (Identical vs. Pseudocha.)	.01	[−.02, .04]	.02	.68	.499
Foveal load × Preview	−.00	[−.06, .06]	.03	−.12	.904
Display monitor (First vs. Second)	−.02	[−.09, .06]	.04	−.46	.643
*Note*. Significant effects are indicated in bold. CI = confidence intervals; Pseudocha. = Pseudocharacter.					
^a^ To make the fixed factors for skipping data (both in Table 3 and Table 4) from a logistic LME model directly interpretable, we transformed the logit values (a binary variable) to probabilities by using the invlogit() in “arm” package in R.					

**Table 4 tbl4:** Fixed Effects Estimates From the (Generalized) Linear Mixed-Effects Models and 95% Confidence Intervals for the Eye Movement Measures at the Target Word

Fixed effect	*b*	CI	*SE*	*t/z*	*p*
Skipping probability					
Foveal load (Low vs. High)	**.45**	**[.41, .49]**	**.08**	**−2.72**	**.007**
Preview (Identical vs. Pseudocha.)	**.36**	**[.32, .39]**	**.08**	**−7.52**	**<.001**
Foveal load × Preview	.49	[.41, .56]	.15	−.37	.711
Display monitor (First vs. Second)	.51	[.43, .58]	.16	.21	.831
First fixation duration					
Foveal load (Low vs. High)	−.01	[−.04, .03]	.02	−.51	.614
Preview (Identical vs. Pseudocha.)	**.10**	**[.07, .14]**	**.02**	**5.20**	**<.001**
Foveal load × Preview	.03	[−.04, .10]	.04	.90	.370
Display monitor (First vs. Second)	−.03	[−.08, .02]	.03	−1.15	.253
Single fixation duration					
Foveal load (Low vs. High)	−.02	[−.06, .02]	.02	−1.03	.306
Preview (Identical vs. Pseudocha.)	**.13**	**[.08, .17]**	**.02**	**5.76**	**<.001**
Foveal load × Preview	.02	[−.05, .09]	.04	.53	.598
Display monitor (First vs. Second)	−.02	[−.07, .03]	.03	−.83	.407
Gaze duration					
Foveal load (Low vs. High)	−.01	[−.05, .03]	.02	−.44	.662
Preview (Identical vs. Pseudocha.)	**.15**	**[.11, .20]**	**.02**	**6.87**	**<.001**
Foveal load × Preview	.01	[−.06, .08]	.03	.33	.739
Display monitor (First vs. Second)	−.01	[−.07, .04]	.03	−.52	.606
*Note.* Significant effects are indicated in bold. CI = confidence intervals, Pseudocha. = Pseudocharacter.					

**Table 5 tbl5:** Probabilities and Mean Saccade Lengths of the Launch and Landing Sites From and to Each of the Regions of Interest as Illustrated in the Example Sentence in Figure 2

Measure	2a→3	2b→3	2a→4a	2b→4a	2a→4b	2b→4b	1→3	1→4a	1→4b
Probability (%)	22.9	24.6	6.5	20.5	2.6	7.0	10.4	3.3	2.1
Saccade length (Character)	1.9	1.2	2.7	1.9	4.4	3.4	3.7	4.6	7.7
*Note.* The arrow represents the direction of the saccade.									

**Table 6 tbl6:** Fixed Effects Estimates From the Linear Mixed-Effects Models and 95% Confidence Intervals for the Eye Movement Measures at the Combined Region of the Target Word and Posttarget Character

Fixed effect	*b*	CI	*SE*	*t*	*p*
First fixation duration					
Foveal load (Low vs. High)	−.02	[−.05, .01]	.01	−1.39	.175
Preview (Identical vs. Pseudocha.)	**.08**	**[.05, .12]**	**.02**	**4.50**	**<.001**
Foveal load × Preview	.03	[−.02, .08]	.03	1.09	.278
Display monitor (First vs. Second)	−.04	[−.09, .01]	.03	−1.39	.167
Single fixation duration					
Foveal load (Low vs. High)	−.02	[−.05, .01]	.02	−1.35	.185
Preview (Identical vs. Pseudocha.)	**.14**	**[.09, .18]**	**.02**	**6.45**	**<.001**
Foveal load × Preview	.02	[−.05, .08]	.03	.48	.630
Display monitor (First vs. Second)	−.03	[−.09, .02]	.03	−1.25	.215
Gaze duration					
Foveal load (Low vs. High)	−.02	[−.06, .02]	.02	−1.00	.323
Preview (Identical vs. Pseudocha.)	**.17**	**[.13, .21]**	**.02**	**7.89**	**<.001**
Foveal load × Preview	.01	[−.06, .08]	.04	.30	.767
Display monitor (First vs. Second)	.00	[−.06, .07]	.03	.13	.900
*Note.* Significant effects are indicated in bold. CI = confidence intervals, Pseudocha. = Pseudocharacter.					

## Footnotes

1 In the current analysis, we removed outliers fixation times based on raw data. We also conducted an analysis of fixation times removing outliers based on log-transformed data. The results showed extremely similar patterns of effects and there were no changes in the effects that were significant across the analyses.
